# Isolation, conventional and molecular characterization of *Salmonella* spp. from newly hatched broiler chicks

**DOI:** 10.1186/s13568-019-0821-6

**Published:** 2019-08-30

**Authors:** Mahmoud E. Sedeik, Nahed A. El-shall, Ashraf M. Awad, Sally M. Elfeky, Mohamed E. Abd El-Hack, Elsayed O. S. Hussein, Abdullah N. Alowaimer, Ayman A. Swelum

**Affiliations:** 1 Department of Poultry and Fish Diseases, Faculty of Veterinary Medicine, Alexandria University, Edfina, Elbehira, 22758 Egypt; 2 Poultry Department, Faculty of Agriculture, Zagazig University, Zagazig, 44511 Egypt; 3 Department of Animal Production, College of Food and Agriculture Sciences, King Saud University, P.O. Box 2460, Riyadh, 11451 Saudi Arabia; 4 Department of Theriogenology, Faculty of Veterinary Medicine, Zagazig University, Zagazig, 44519 Egypt

**Keywords:** *Salmonella*, Chicken, Bactreiolgical, ERIC-PCR, Serotypes

## Abstract

*Salmonella* is an important pathogen for poultry production as well as for human due to zoonotic importance. It has more than 2600 identified serovars despite of this identification and classification of *Salmonella* isolates into different serovars is critical for study of incidence and surveillance. This study investigates the epidemiology and molecular characterization of *Salmonella* isolates in broiler chicks during 1st week of life. A total of (n = 1000) samples including liver, intestine, yolk sac, spleen and heart blood were collected from El-Gharbia, El-Behera, Kafr-Elshikh, Alexandria, Marsamatroh Provinces in Egypt and tested through bacteriological, biochemical, serological and molecular examinations. Incidence of *Salmonella* was demonstrated on 75 positive samples from 1000 samples and the predominance of *Salmonella* that *i*solated from internal organs of newly hatched chicks was highest from yolk sacs (10%), liver and intestines (9%) followed by the spleen (7.5%) then heart blood (2%). Serotyping of the isolated strains using slide agglutination test revealed that 24 isolates belonging to *S. enteritidis* (1,9,12 g.m 1,7), while, 14 isolates belonging to *S. virchow* (6,7 r 1,2), in addition to, 12 isolates belonging to *S. typhimurium* (1,4,5,12.i.1,2) and 8 isolates belonging to *S. kentucky* (6,8.I,z). Enterobacterial Repetitive Intergenic Consensus (ERIC) PCR revealed that two *S. enteriditis* isolates were identical and one isolate differ by 40%, while two *S. typhimurium* isolates were identical by 80% and one isolate was similar by 20% to the other two isolates, in addition, two *S. virchow* isolates were identical by 80% and the two *S. kentucky* isolates were different.

## Introduction

*Salmonella* isolates are considered as the most circulating and frequent bacterial agents causing disease poultry and other avian species. It is associated with high economic losses because of high mortality, morbidity and impaired productions. It is considered as a major food-borne pathogen in most countries of the world especially in developing countries **(**Soultose et al. 2003; Carraminana et al. 2004). *Salmonella* contamination of poultry and poultry products are frequently occurred and can be transmitted to humans through transportation and consumption of undercooked poultry meat (Bailey and Cosby 2003; Kimura et al. 2004). Wide variations of *Salmonella* serovars commonly infect poultry and one serovar may be common in a country for number of years before it is substituted by another isolate. The serovars may vary geographically, but the most common serovars reported globally are *S. typhimurium* and *S. enteritidis* as reported by World Health Organization (2006). Salmonellosis has been associated with infection of broiler flocks that has ability of vertical transmission to progeny (Irshad et al. 2013). The predominant serotypes have been identified in Egyptian poultry farms are *Salmonella enterica* serovar *Typhimurium* and *S. enterica* serovar *Enteritidis* (Abd El-Ghany et al. 2012).

Serotyping is a basic biomarker to investigate the epidemiological situation of *Salmonella* infections and it is commonly used to trace back the contamination sources during outbreaks. White and Kauffmann developed the serotyping scheme on 1920 that was based on the flagella H, somatic O antigens and the observed phase-shift in flagella antigen (Molbak et al. 2006). This method is worldwide and it is considered as the standard method for *Salmonella* serotypes identification. The advantages of identifying *Salmonella* serotypes include providing information about the disease severity, contamination source and the resistance pattern (Molbak et al. 2006). Moreover, molecular techniques have been used to differentiate the strains of *Salmonella* isolates including pulsed field gel electrophoresis (PFGE), enterobacterial repetitive intergenic consensus (ERIC) PCR, Random Amplification of Polymorphic DNA (RAPD), Single Strand Conformation Polymorphism (SSCP), hybridization and ribotyping-PCR (Anjay et al. 2015). Due scarce knowledge available on conventional and molecular identification of *Salmonella* species, this investigation was designed to follow the epidemiology of *Salmonella* isolates through biochemical, serological and molecular methods.

## Materials and methods

### Sample collection

A total of one thousand samples including liver, intestine, yolk sac, spleen and heart blood of newly hatched chicks during first week of life were collected aseptically from 25 poultry farms located in five different governorates in Egypt (El-Gharbia, El-Kafr-Elshikh, El-Behera, Alexandria and Matroh) with 10 chicks for each farm as shown in Table 1. The samples were collected in separate sterile plastic bags and immediately transported to the laboratory in ice box (4 °C).

**Table 1:** History of examined farms

Farm No.	Location	No. of chicks	Total farm No.	Age of chick (day)	Mortalities in week %	Antibiotic used at 1st 3 days of age
1	El-Gharbia	40	5000	1	10	Ciprofloxacin
2	El-Gharbia	40	7000	5	15	Colistine + tylosine
3	El-Gharbia	40	5000	3	5	Ciprofloxacin
4	El-Gharbia	40	10,000	5	12	Florfenicol
5	El-Gharbia	40	12,000	1	18	Ciprofloxacin
6	El-Behera	40	15,000	3	15	Enrofloxacin + colistine
7	El-Behera	40	5000	1	2	Enrofloxacin
8	El-Behera	40	10,000	5	7	Colistine + tylosine
9	El-Behera	40	15,000	3	12	Ciprofloxacin
10	El-Behera	40	20,000	1	14	Florfenicol
11	Kafr-Elshikh	40	10,000	5	10	Enrofloxacin
12	Kafr-Elshikh	40	10,000	5	8	Oxytetracyclin + tylosine
13	Kafr-Elshikh	40	15,000	3	7	Enrofloxacin + colistine
14	Kafr-Elshikh	40	20,000	1	10	Ciprofloxacin
15	Kafr-Elshikh	40	10,000	3	5	Florfenicol
16	Alexandria	40	15,000	5	8	Ciprofloxacin
17	Alexandria	40	15,000	5	14	Colistine + tylosine
18	Alexandria	40	5000	1	5	Ciprofloxacin
19	Alexandria	40	5000	5	10	Ciprofloxacin
20	Alexandria	40	10,000	1	15	Oxytetracyclin + tylosine
21	Marsamatroh	40	5000	1	5	Oxytetracyclin + tylosine
22	Marsamatroh	40	5000	5	12	Enrofloxacin
23	Marsamatroh	40	10,000	1	20	Ciprofloxacin
24	Marsamatroh	40	5000	1	7	Florfenicol
25	Marsamatroh	40	5000	3	2	Ciprofloxacin

### Bacterial isolation

The collected samples were cultured on 1% peptone broth then 1 ml selenite F. broth and incubated aerobically at 37 °C for 18 h then were subcultured to MacConkey, *Salmonella shigella* agar and/or XLD media. The cultured plates were incubated at 37 °C for 24 h. Suspected colonies were picked up, preserved into semi solid agar as stock medium and into slant agar for further biochemical and serological identification.

### Biochemical identification

Dry heat fixed smears of suspected colonies were stained using Gram’s stain then were examined, revealing the presence of Gram negative bacilli. The suspected isolates were identified biochemically (Hossain et al. 2006) by applying catalase test, oxidase test and IMViC group of biochemical tests. The identified isolates as *Salmonella* species were cultivated on triple sugar iron agar (TSI).

### Serological identification

Serogrouping of identified bacterial isolates was performed according to Kauffmann–White method (Aribam et al. 2015).

### Molecular identification

Biochemically, identified *Salmonella* isolates were then serotyped and further characterization was done by using ERIC PCR for intra-serotyping of *Salmonella* isolates. DNA was extracted from studied isolates according to QIAamp DNA mini kit instructions and PCR Master Mix was prepared according to Emerald Amp GT PCR master mix (Tarkara) Code.No.RR310Akit using the following primer set ERIC-DG111-F with primers sequences ATG TAA GCT CCT GGG GAT TCA C and ERIC-DG112-R with primers sequences AAG TAA GTG ACT GGG GTG AGC G. Amplification of primers was done by using thermal cycling (Fendri et al. 2013). Briefly, denaturation at 94 °C for 2 min, annealing at 49 for and extention at 72 for 2 min followed by 35 cycles including 94 °C for 1 min, 56 °C for 1 min and 72 °C for 2 min and final extension at 72 °C for 5 min. After that the amplified product was loaded on 1.5% agrose gel using 100 bp gene ruler for 1 h at 5 V and the gel was visualized by chemical documentation (Bio Red).

## Results

### Morphological identification of the isolated organisms

Morphology revealed the 75 samples out of one thousands appeared on MacConkey agar, colorless and translucent, though they sometimes have dark centers. Gram’s stain smears from suspected colonies showed Gram-negative rod-shaped motile bacteria, or bacillus. On XLD, they were pink with or without black centers while, colonies on S.S agar media appeared as white colonies with black center.

### Biochemical identification of the isolated organisms

The isolated micro-organisms were positive for methyl red, catalase, TSI, citrate utilization test, lysine iron agar, oxidase and christensen citrate while negative to indole, Phenol red, sucrose, and Voges-Proskauer. They ferment variety of sugar types but remain negative on KCN medium and ONPG reaction as illustrated in Table 2.

**Table 2:** Biochemical identification of various organisms suspected to *Salmonella* isolates

Biochemical tests	*Salmonella* isolates
Indole	−ve
Methyl red	+ve
Voges Proskauer	−ve
Citrate utilization test	+ve
TSI	K/A. + ve H2S
Lysine iron agar	+ve
Christensen citrate	+ve
Hydrolysis of urea	−ve
Gelatin liquefaction	−ve
Oxidase test	−ve
Ornithine decarboxylase	+ve
Mannitol	+ve
l-arabinose	+ve
Maltose	+ve
l-rhamnose	+ve
Glucose	+ve
KCN medium	−ve
ONPG-reaction	−ve
Catalase test	+ve

### Incidence of *Salmonella* in different organs

Revealing to traditional identification on media and biochemically identification the proportion of isolates result as *Salmonella* isolates from various organs of newly hatched chicks represented by 7.5% total distribution in various organ as shown in Table 3.

**Table 3:** Incidence of *Salmonella* isolates in various organs of 1 week old chicks

Organs	No. of examined organs	No of *Salmonella* +ve organs	Percentage (%) of isolation
Liver	200	18	9
Yolk sac	200	20	10
Intestine	200	18	9
Spleen	200	15	7.5
Heart blood	200	4	2
Total	1000	75	7.5

### Serological identification of the isolated organisms

The serotyping investigated the *S. typhimurium*, *S. enteritidis*, *S. virchow* and *S. kentucky* with O antigen are 4, 3, 2 and 2 while presence of H factor only in *S. enteritidis. S. typhimurium and S. virchow* as shown in Table 4.

**Table 4:** Results of serotyping of the isolated *Salmonella* strains

Serial No.	*Salmonella* serotype	Group	Antigenic structure		
			O-antigen	H-factor	
				Phase I	Phase II
1	*S. enteritidis*	D	1,9,12	g,m	1.7
2	*S. typhimurium*	B	1,4,5,12	I	1.2
3	*S. virchow*	C1	6,7	R	1.2
4	*S. kentucky*	C3	6,8	L.z	–

### Strain wise distribution of *Salmonella* species

Serotyping revealed that the distribution of *S. entritidis* was comparatively higher than *S. Virchow, S. typhimurium* and *S. Kentucky* as 2.4, 1.4, 1.2 and 0.8% while 1.7% strains were untypable as illustrated in Table 5.

**Table 5:** Strain wise distribution of isolated *Salmonella* species

*Salmonella* serotype	No. of the isolated strains	% of the isolated strains
*S. enteritidis*	24	2.4
*S. typhimurium*	12	1.2
*S. Virchow*	14	1.4
*S. Kentucky*	8	0.8
Un typable	17	1.7
Total isolated strains	75	7.5

**Fig. 1:**
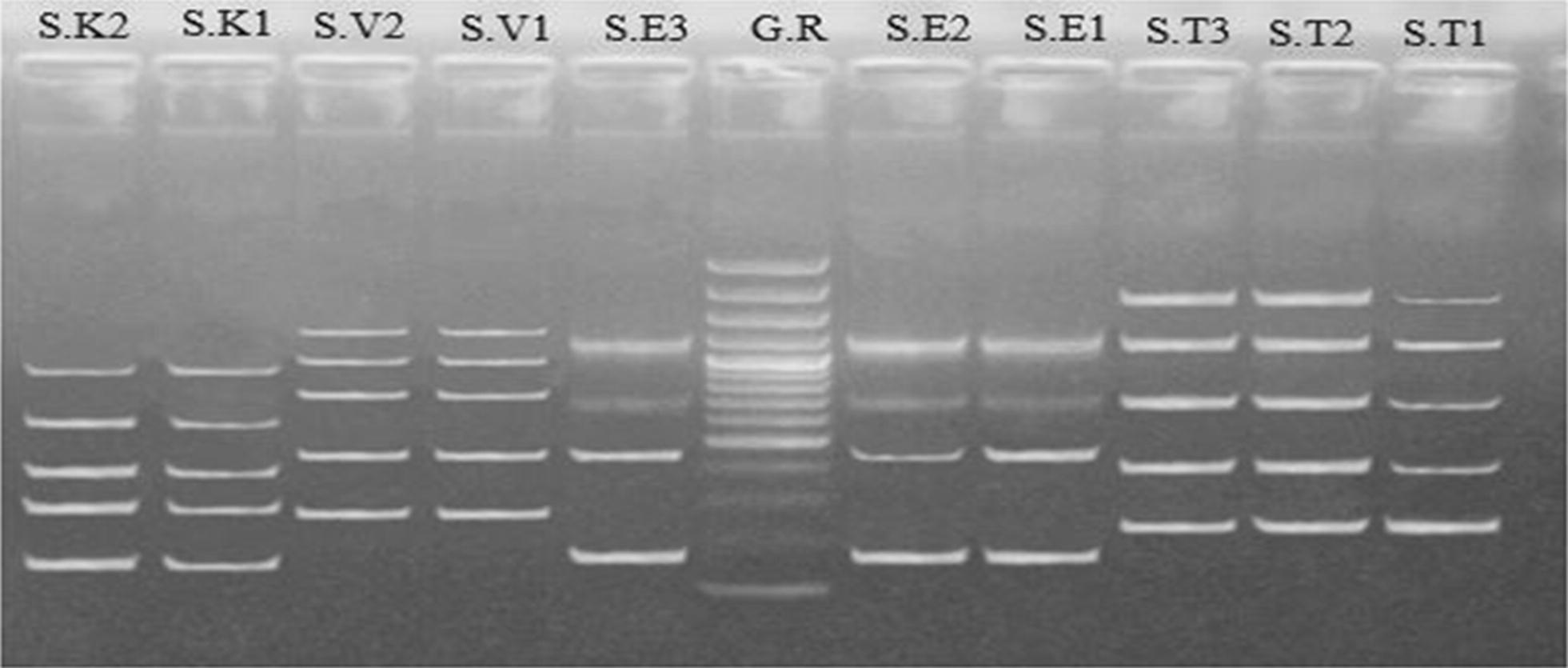
ERIC PCR of different isolates of *Salmonella.* lane S. T1, S. T2 and S. T3 are *S. typhimurium*, lane S.E1, S.E2 and S.E3 are *S. enteritidis,* lane S.V1 and S.V2 are *S. virchow* while lane S.K1 and S.K2 are *S. Kentucky* verified by using lane G.R 100 bp gene ruler

ERIC-PCR revealed that two *Salmonella enteritidis* were found identical while one was different i-e; lane S. T1, S. T2 and S. T3 with 232, 235 and 235 bp respectively. Similarly, three *S. typhimurium* were identical i-e; lane S.E1, S.E2 and S.E3 166, 166 and 166 bp. Additionally, lane S.V1 and S.V2 i-e; 266 and 266 bp showed two *S. virchow* were identical to each other while lane S.K1 and S.K2 i-e; 149 and 151 bp revealed that two *S. Kentucky* differ from each other as shown in Table 6 and Fig. 1.

**Table 6:** ERIC PCR for selected strain of investigated *Salmonella*

PCR bands (bp)									
S.T1	S.T2	S.T3	S.E1	S.E2	S.E3	S.V1	S.V2	S.K1	S.K2
1909	1968	1938	1200	1200	1200	1372	1357	958	948
1200	1214	1214	1070	1070	1070	1034	1034	576	581
700	700	700	708	700	700	760	760	378	378
391	395	395	450	445	445	445	445	277	280
232	235	235	166	166	166	266	266	149	151

## Discussion

*Salmonella* is considered as one of the major pathogenic agents which infect the variety of avian species specially poultry birds including layer as well as broiler reared in the modern intensive system with higher biosafety, biosecurity and standard management. Any contributions for elimination of *Salmonella* incidences and infection in birds could have a major influence in reducing the populations of the organism under natural conditions. One thousand samples were collected from different farms including liver, intestine, yolk sac, spleen and heart blood of newly hatched chicks at El-Gharbia, El-Behera, Kafr-Elshikh, Alexandria, Marsamatroh Provinces. The samples were examined bactriologically to isolate the *Salmonella* isolates.

In this study, 75 samples out of 1000 samples (7.5%) were found positive. The higher percentage of isolation from the internal organs from yolk sacs (10%) then from livers (9%) and from 20 intestines (9%) were the same, spleen (7.5%), and finally heart blood (2%) (Table 3). These results are in contrary to (Ahmed et al. 2008; Islam et al. 2016) who showed that the prevalence of avian Salmonellosis was highest in adult layer (53.25%) followed by brooding (14.55%) then growing (16.10%) and pullet (16.10%). The prevalence rate of *Salmonella* spp. in different poultry farm were different i-e; the 80 samples were tested from the clinically healthy birds showed 44 (55%) positive (Ahmed^14^). Moreover the samples from birds having diarrhea infection rate (66.67%) (Hossain et al. 2006).

The study revealed that pink colonies with or without black centers were typical for *Salmonella* on XLD. Many cultures of *Salmonella* spp. may produce large colonies with glossy black centers or may appear as almost completely black that is similar to (Ramya et al. 2012). Correspondingly, the characteristics of *Salmonella* spp. colonies are translucent, small round, smooth, black or colorless was observed on SSA, black colonies on TSI agar (Islam et al. 2016 and Sujatha et al. 2003). The isolated micro-organisms were Catalase-positive, oxidase, indole, Phenol red, sucrose, Voges-Proskauer and urease negative while methyl red, H2S production, citrate-positive and glucose positive. The current finding is similar Islam et al. (2016) who have found *Salmonella* isolates were MR test and citrate utilization test positive, ferment dextrose, maltose and mannitol but fail to ferment sucrose and lactose.

In the present study serological identification of the isolated bacteria revealed 24 isolates belonging to group D and identified as *S. enteritidis* (1,9,12. g,m 1,7) and 12 isolates belonging in the group B and identified as *S. typhimurium* (1,4,5,12.i.1,2) and 14 isolates belonging in the group C1 and identified as *S. virchow* (6,7.r,1,2) and 8 isolates belonging in the group C3 and identified as *S. kentucky* (6,8.I,z). Meanwhile, 17 isolates were untypable (Table 4). Moreover, 68 serotypes were identified among 75 *Salmonella* isolates, and 17 isolates were untypeable (Table 5). The most prevalent serovar detected in this study was *S. enteritidis* 2.4% followed by *S. virchow* 1.4%, *S. typhimurium* 1.2% and *S. kentucky* 0.8%. The most commonly isolated serotype from different organs was *S. enteritidis* the same results were recorded in Egypt by (Sujatha et al. 2003; Akeila et al.^2013^ and Rabie et al. 2012) who confirmed the prevalence of *S. enteritidis* and *S. typhimurium* by (58.33% and 41.66%), respectively from chickens. In addition, *S. enteritidis* and *S. typhimurium* were predominant in Saudi Arabia, by (55.56% and 22.22%, respectively) among the detected *Salmonella* serovars from chickens (Moussa et al. 2010). Im et al. (2015) reported that the most prevalent *Salmonella* serovars in the flocks were *Salmonella bareilly* 41.2%), *Salmonella mbandaka* (32.4%), and *Salmonella rissen* (17.6%).

Ten *Salmonella* isolates belonging to 4 serotypes produced ERIC PCR fingerprints that were distinct for each serotype (Table 6). ERIC PCR found that three *S. enteriditis* isolates (isolates 2 and 3 identical in 1200, 1070, 700, 445, 166 bands but isolate 1 different from it in 708,450 bands) so two *S. enteriditis* isolates were identical and one isolate was different from it by 40%. Three *S. typhimurium* isolates (isolate 1 belonging to 1909, 1200, 700, 391 and 232 bands it was different from isolates 2 and 3 while isolates 2 and 3 identical in 1214, 700, 395, 235 bands and the difference in 1968 and 1938 bands) so two *S. typhimurium* isolates were identical by 80% and one isolate was similar by 20% to the other two isolates two *S. virchow* isolates were identical in 1034, 760, 445, 266 bands and the difference in 1372 and 1357 bands) so two *S. virchow* isolates were identical by 80%. Two *S. kentucky* isolates (isolate 1 belonging to 958, 576, 378, 277, 149 bands and it was different from isolates 2 which belonging to 948,581,378,280,151 bands) so two *S. kentucky* isolates were not identical. ERIC-PCR is a useful and recent method for DNA typing for analysis and evaluation of fingerprinting. It is used in epidemiology of *Salmonella enteritidis* (Suh and Song 2006). Using specific ERIC primers, a total of 30 strains of *Salmonella enteritidis* of four main clusters had found 60% similarity.

This study found that the Serotyping of the isolated strains revealed that 24 isolates belonging to *S. enteritidis* (1,9,12 g.m 1,7), while, 14 isolates belonging to *S. virchow* (6,7 r 1,2), in addition to, 12 isolates belonging to *S. typhimurium* (1,4,5,12.i.1,2) and 8 isolates belonging to *S. kentucky* (6,8.I,z). ERIC-PCR revealed that two *S. enteriditis* isolates were identical and one isolate was different from it by 40%, while two *S. typhimurium* isolates were identical by 80%and one isolate was similar by 20% to the other two isolates, in addition to, two *S. virchow* isolates were identical by 80% and *the* two *S. kentucky* isolates were not identical. This study will help future researchers to uncover new and critical methods that should be used to improve diagnosis and control measures for prevention zoonotic infections of *Salmonella* species.

## Data Availability

Not applicable.
